# [Retracted] Salvianolic acid B protects against sepsis-induced liver injury via activation of SIRT1/PGC-1α signaling

**DOI:** 10.3892/etm.2025.12959

**Published:** 2025-08-28

**Authors:** Hongling Su, Zhisheng Ma, Aixia Guo, Hong Wu, Xiangmin Yang

Exp Ther Med 20:2675–2683, 2020; DOI: 10.3892/etm.2020.9020

Following the publication of this paper, it was drawn to the Editors’ attention by a concerned reader that the western blot data for the protein Bax in Fig. 5C on p. 2679 were strikingly similar to western blot data that had already been accepted for publication in different form in another article submitted to *Journal of Neurochemistry* that had been written by different authors at different research institutes.

In view of the fact that the abovementioned data had already apparently been published previously, the Editor of *Experimental and Therapeutic Medicine* has decided that this paper should be retracted from the Journal. The authors were asked for an explanation to account for these concerns, but the Editorial Office did not receive a reply. The Editor apologizes to the readership for any inconvenience caused.

